# Strategies to Build Observation Skills for Integrative and Organismal Biology Undergraduates: A Scoping Review

**DOI:** 10.1093/iob/obag037

**Published:** 2026-07-13

**Authors:** Brandi Goss, Kelly Goedde-Matthews, Robert E Furrow

**Affiliations:** Department of Wildlife, Fish and Conservation Biology, University of California, Davis, CA 95616, USA; Department of Wildlife, Fish and Conservation Biology, University of California, Davis, CA 95616, USA; Department of Wildlife, Fish and Conservation Biology, University of California, Davis, CA 95616, USA

## Abstract

The ability to observe natural phenomena and connect those observations to theoretical concepts and processes is critical for knowledge integration and scientific inquiry in integrative and organismal biology. However, no published effort had reviewed evidence-based pedagogical methods for teaching scientific observation skills in integrative and organismal biology. This review found 53 papers with relevant pedagogical interventions for increasing observation skills from a diversity of disciplines (e.g., ecology, health sciences, and environmental sciences) and participant populations (preschool through postgraduate). However, only 2 studies assessed interventions aimed at improving observation skills for undergraduate biology students. More research is needed to understand how these pedagogical strategies might translate to undergraduate integrative and organismal biology students. Pedagogical strategies fell into 4 main categories (manipulating the learning environment, scaffolding the observation process, and engaging with arts interpretation or creation), with arts interpretation showing particular promise for translation to integrative and organismal biology applications.

## Introduction

Observation skills are necessary for meaningful inquiry in a range of scientific fields (Haury 2002; Eberbach and Crowley 2009; Wellbery and McAteer 2015; Cheatham 2023). However, there is no consensus definition of observation across scientific disciplines, and the crucial aspects of an effective observation vary by discipline. For example, students on a biology field trip may aspire to make insightful field notes from observations of wild animals, while a health professional’s goal may be to correctly diagnose a medical condition by observing a patient’s signs and symptoms. Eberbach and Crowley (2009) outline a broadly applicable observation framework with four dimensions of the observation process: noticing, expectations, observation records, and productive dispositions. Noticing describes the process of identifying relevant features and ignoring irrelevant ones, while situating the observation within a broader organizational framework of related phenomena. Expectations describe an observer’s ability to connect observations to theoretical concepts for deeper inference. Observational records are the tools and habits used to create a record of observations, and productive dispositions refer to how an observer’s interests and identity drive their observation practices. Within each of these dimensions, observations can range from simple “everyday” observations to intentional “scientific” observations. An everyday observation may include a superficial mix of relevant and irrelevant features with little connection to broader knowledge or concepts and no effort to record, while a scientific observation focuses on key features and connects to both prior knowledge of the observed system and to interconnected theories that generate new inferences and questions (Eberbach and Crowley 2009).

The ability to observe natural phenomena is a core part of scientific reasoning in integrative and organismal biology. Indeed, the “formal practices of observation” are central to applying the process of science (American Association for the Advancement of Science 2011) and can even support student learning of quantitative biology material as students use real-world observation to develop intuition about mathematical relationships (National Research Council 2003). Organismal biologists often rely on the observation of living things or their preserved specimens, for example, when studying vertebrate anatomy (Danos et al. 2022). Many organismal biology courses already include an emphasis on observation for the purpose of species identification or ecological interpretation, offering opportunities for instructors to implement evidence-based teaching practices to cultivate observation skills without requiring substantial overhauls to existing courses. To that end, a review of the literature addressing evidence-based pedagogical tools for improving observation skills is valuable for organismal biology education.

Research on how to teach scientific observation skills is spread across a range of disciplines and includes many potential pedagogical approaches. For example, health sciences students who engage in fine art interpretation (e.g., the Visual Thinking Strategies curriculum; Yenawine 2013) can improve both noticing and expectations during the process of diagnosing patients (Cerqueira et al. 2023). In the natural environment, university students who record field observations in nature journals report an increase in confidence in their observation skills and increased connectedness to nature (Zakariya et al. 2025). Other pedagogical approaches focus on scaffolding the observation process, for example, with a digital dichotomous key for the identification of bird museum specimens (Knight and Davies 2016) or by modeling the eye-gaze patterns of medical experts during diagnosis (Jarodzka et al. 2012). Although several of these approaches have primarily been applied in other disciplines, observation skills in fields as diverse as design, health sciences, and physical sciences may translate to observation in biology contexts. Many such studies can offer insight to the teaching of integrative and organismal biology, but we found no prior effort to synthesize and summarize the observation skills pedagogy literature in this context.

To identify potential pedagogical strategies to teach observation skills in integrative and organismal biology, we conducted a scoping review focused on evidence-based studies that presented both the details of a pedagogical method and some measurement of an outcome related to noticing, expectations, observational records, or productive dispositions. The review included studies from any academic discipline if they met these criteria.

## Materials and methods

We employed a scoping review methodology to identify relevant studies, with no restrictions on discipline or student age. Scoping reviews aim to quickly identify the types and extent of relevant research literature to allow a summary of core research directions, but they do not purport to exhaustively summarize research findings nor fully appraise the quality of each study (Grant and Booth 2009).

### Research question

Although we initially intended to review studies featuring teaching and learning methods for biology undergraduates, we discovered potentially relevant studies across multiple disciplines, with participants ranging from prekindergarten children through postgraduate students. With the goal of finding the broadest possible swath of relevant research, we finalized our research question as: *What pedagogical practices can potentially build observation skills for undergraduate students studying integrative or organismal biology?*

### Identifying relevant studies

We selected data queries through an iterative process of trying queries, discussing the breadth of results, and comparing the results from different databases. Results had limited variation among relevant databases, so we ultimately settled on combining the results of three separate queries to the Web of Science Core Collection (Table S1). Each query attempted to find articles with content about observation skills, a connection to a scientific discipline, and a focus on measuring learning. The initial search on January 28, 2026, produced a total of 954 references.

### Study inclusion criteria

To generate the final study list, we first removed any duplicate references, resulting in 716 references. Next, we reviewed manuscript titles and abstracts to exclude studies that clearly did not focus on education or observation skills, ending with 91 potential studies of interest. Finally, we read these 91 publications and applied inclusion criteria about study methods and result to ensure that all studies offered actionable teaching insight. We included a study in the final data set only if it described a replicable learning experience for students and systematically measured outcomes related to observation skills. A total of 53 studies met these inclusion criteria.

### Charting the data

With our final set of studies, each author independently read three studies and reviewed the abstracts of the full final study set, seeking to clarify the broad dimensions of study information to systematically summarize during the literature review. We identified the following key dimensions: participant characteristics (e.g., student age, discipline, and study country), data collection type (quantitative, qualitative, or mixed-methods), study methodology (single measurement versus repeated measures, presence or absence of control/comparison groups), category of pedagogical method assessed, and type of outcomes measured. We iterated through the precise boundaries of the pedagogical method categories, with authors attempting to apply the categorizations, honing the categories through discussion, then repeating the process until we reached consensus. We ultimately categorized studies based on whether they assessed: the learning environment in which students made observations, strategies for scaffolding or supporting the observation process, arts-based interventions focused on art interpretation to develop observation skills, or arts-based interventions focused on the creation of art (e.g., sketching, creative writing) during the observation process.

## Results

**Fig. 1:**
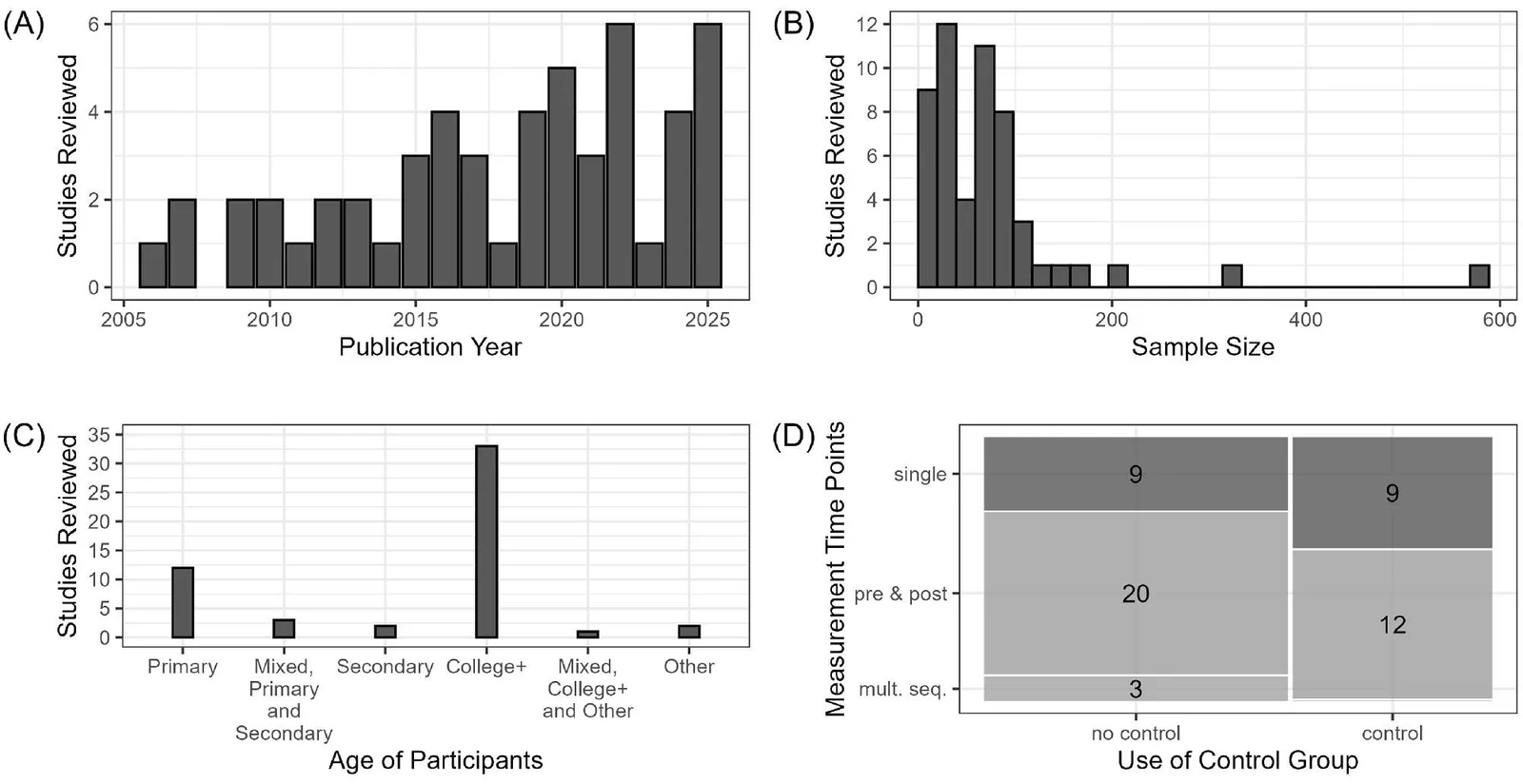
Characteristics of reviewed studies, including: (**A**) frequency of publication year, (**B**) frequency of sample sizes, (**C**) frequency of studies addressing specific age groups, and (**D**) a mosaic plot of the number of measurements (single, pre and post, or multiple sequential) and use of control groups in the reviewed studies.

Our initial queries identified 954 publications; we ultimately included 53. The average publication year of the included studies is 2018, with most papers being published between 2015 and 2025 (Fig. 1A).

### Disciplines

The majority of reviewed studies come from health fields, with most addressing training in fine arts interpretation to support observation skills in diagnostic assessments (24; Table 1, e.g., Monahan et al. 2019; Slota et al. 2022; Yucel et al. 2025). More recently, the use of observation skills to support diagnostic processes has been studied in dentistry and veterinary medicine (Beck et al. 2017; Fernandez et al. 2021, 2022; Wolf et al. 2024; Qutieshat 2025). Additionally, approaches to scaffold the observation process have been applied in medical diagnostic analysis such as eye-tracking and feature spotlighting, with documented improvements in observation of relevant diagnostic features (e.g., Jarodzka et al. 2012; Suzuki and Kurita 2025 with less conclusive results). Finally, at least two studies assessed the influence of lessons using a simulated hospital room to support observation skill learning (Yoneda et al. 2017; Reime et al. 2022).

**Table 1:** Number of studies included in this scoping review in each of the following disciplines with associated references.

**Discipline**	**Number of included studies**	**References**
Health fields (nursing, medicine, psychology, physiotherapy)	24	(Agarwal et al. 2020; Alenezi 2020; Chen et al. 2025; Cole et al. 2020; Ferrara et al. 2022; Ghorbani et al. 2025; Guo et al. 2021; Gurwin et al. 2018; He et al. 2019; Jarodzka et al. 2012; Jasani and Saks 2013; Kirklin et al. 2007; Klugman et al. 2011; Lyon et al. 2013; Monahan et al. 2019; Pellico et al. 2009; Poirier et al. 2020; Reime et al. 2022; Shapiro et al. 2006; Slota et al. 2022; Suzuki and Kurita 2025; Wulfeck et al. 2024; Yoneda et al. 2017; Yucel et al. 2025)
Dentistry	1	(Qutieshat 2025)
Veterinary science	4	(Beck et al. 2017; Fernandez et al. 2021, 2022; Wolf et al. 2024)
Design	2	(Zakariya et al. 2025) (landscape design) and (Park and Logsdon 2015) (3D models)
Teacher training	1	(Star and Strickland 2008)
Biology/Ecology	15	(Boerwinkel et al. 2009; Colucci-Gray et al. 2025; Donald and Barker 2016; Eberbach and Crowley 2017; Hung et al. 2010, 2012; Hwang et al. 2010; Klofutar et al. 2022; Knight and Davies 2016; Kos et al. 2015; Louw and Sanford-Dolly 2024; Race et al. 2021; Tanaka et al. 2016; Wyner and Doherty 2021; Yurumezoglu and Oztas Cin 2019)
Astronomy	2	(Cole et al. 2015; Zhang et al. 2014)
Hydrology	1	(Lechner et al. 2024)
Geology	2	(Choi and Kim 2022; Frøyland et al. 2016)
Chemistry	1	(Terra and Black 2019)

Biology and Ecology disciplines had the second largest number of studies in our review (15; Table 1). These studies focused almost exclusively on prekindergarten—secondary school children (e.g., Boerwinkel et al. 2009; Hung et al. 2010; Wyner and Doherty 2021; but see: Race et al. 2021; Zakariya et al. 2025). Interestingly, despite having fewer publications in our review, these studies covered a wider range of intervention types and their effects on observation skill acquisition. These included different learning environments (online vs. in-person; Race et al. 2021), observation process scaffolding and support tools such as concept maps (Hung et al. 2012) and digital tools (Hung et al. 2010; Louw and Sanford-Dolly 2024), and art creation as a tool for processing and communicating observations (Donald and Barker 2016). However, visual thinking strategies, a common framework used in the health sciences literature for increasing observation skills, was not present in the biology and ecology literature.

Several papers come from the physical and environmental sciences such as astronomy, hydrology, geology, and chemistry (Table 1). These studies address observation support tools such as artificial reality tools that reduce environmental (e.g., cloud cover) and traditional tool (e.g., difficulties focusing a telescope) limitations in astronomy (Zhang et al. 2014) and toys with observable chemical phenomena (Terra and Black 2019). Other studies also implement education about the observation process to improve student observation skills for rock identification (Frøyland et al. 2016; Lechner et al. 2024). Studies in these disciplines notably did not explicitly investigate art-based approaches, although one study used moon journals which included the use of writing and drawing skills to improve observation of lunar phases and spatial-scientific reasoning (Cole et al. 2015).

The remaining three studies come from landscape design (Zakariya et al. 2025), model design (Park and Logsdon 2015), and teacher training (Star and Strickland 2008). Zakariya et al. (2025) addresses the use of nature journaling to advance student observation skills. Star and Strickland (2008) focus on observation processes such as guiding questions and video review to enhance the quality of observations. Park and Logsdon (Park and Logsdon 2015) investigate how 3D model building supports written communication of observed steps in an assembly process.

### Study populations

The sample size in the reviewed studies ranged from four to 573 (Fig. 1B), with a mean sample size of 75 (median = 62, standard deviation (SD) = 89.2). Studies ranged from teaching observation skills at the prekindergarten level to the college and professional school level (Fig. 1C). For the sake of our review, we defined primary school as anything before 7th grade, secondary school as 7th grade through high school, and college + as college, graduate school, and professional school (e.g., medicine, veterinary medicine, teaching). The “other” group included two studies conducted outside of school environments with volunteers for a water quality training program (Louw and Sanford-Dolly 2024) and medical practitioners and nurses (Kirklin et al. 2007).

### Experimental designs

The 53 studies reviewed contained a mixture of quantitative, qualitative, and mixed experimental methods (Table 2). Fewer than half of the studies reviewed compared a treatment group which received the intervention to a control group which did not receive the intervention (Table 2 and Fig. 1D). This finding is consistent with a recent review of the literature on visual thinking strategies training in medical fields (Cerqueira et al. 2023) which similarly found a lack of controlled studies. Additionally, one-third of the studies included only a single assessment of outcomes (Table 2 and Fig. 1D). Many of these studies assessed students’ perceptions about their own observation competency and motivation, or were qualitative in nature (e.g., Beck et al. 2017; Eberbach and Crowley 2017; He et al. 2019). However, the majority of the studies (32) contained both pre-intervention and post-intervention measurements, allowing assessment of the change in a student’s observation skills as a result of the intervention (e.g., Klugman et al. 2011; Fernandez et al. 2022; Slota et al. 2022). Notably, 13 studies had both a control group and measured response variables before and after the intervention (Fig. 1D; Kirklin et al. 2007; Zhang et al. 2014; Knight and Davies 2016; Gurwin et al. 2018; Agarwal et al. 2020; Guo et al. 2021; Race et al. 2021; Wyner and Doherty 2021; Ferrara et al. 2022; Klofutar et al. 2022; Lechner et al. 2024; Ghorbani et al. 2025; Yucel et al. 2025).

**Table 2:** Number of studies included in this scoping review with each of the following measurement approaches and associated references.

**Measurement approach**	**Number of included studies**	**References**
Quantitative research methods	27	(Alenezi 2020; Chen et al. 2025; Cole et al. 2020; Cole et al. 2015; Fernandez et al. 2021, 2022; Ferrara et al. 2022; Ghorbani et al. 2025; Guo et al. 2021; Gurwin et al. 2018; Hung et al. 2012; Hwang et al. 2010; Jarodzka et al. 2012; Kirklin et al. 2007; Klofutar et al. 2022; Klugman et al. 2011; Lechner et al. 2024; Monahan et al. 2019; Park and Logsdon 2015; Reime et al. 2022; Slota et al. 2022; Star and Strickland 2008; Terra and Black 2019; Wulfeck et al. 2024; Yoneda et al. 2017; Yucel et al. 2025; Zhang et al. 2014)
Qualitative research methods	12	(Boerwinkel et al. 2009; Choi and Kim 2022; Donald and Barker 2016; Eberbach and Crowley 2017; Frøyland et al. 2016; He et al. 2019; Jasani and Saks 2013; Lyon et al. 2013; Pellico et al. 2009; Shapiro et al. 2006; Tanaka et al. 2016; Yurumezoglu and Oztas Cin 2019)
Mixed research methods	14	(Agarwal et al. 2020; Beck et al. 2017; Colucci-Gray et al. 2025; Hung et al. 2010; Knight and Davies 2016; Kos et al. 2015; Louw and Sanford-Dolly 2024; Poirier et al. 2020; Qutieshat 2025; Race et al. 2021; Suzuki and Kurita 2025; Wolf et al. 2024; Wyner and Doherty 2021; Zakariya et al. 2025)
Control group included	21	(Agarwal et al. 2020; Beck et al. 2017; Chen et al. 2025; Eberbach and Crowley 2017; Ferrara et al. 2022; Ghorbani et al. 2025; Guo et al. 2021; Gurwin et al. 2018; Jarodzka et al. 2012; Kirklin et al. 2007; Klofutar et al. 2022; Knight and Davies 2016; Kos et al. 2015; Lechner et al. 2024; Pellico et al. 2009; Qutieshat 2025; Race et al. 2021; Shapiro et al. 2006; Wyner and Doherty 2021; Yucel et al. 2025; Zhang et al. 2014)
Pre-post measurements	32	(Agarwal et al. 2020; Alenezi 2020; Cole et al. 2015; Fernandez et al. 2021, 2022; Ferrara et al. 2022; Ghorbani et al. 2025; Guo et al. 2021; Gurwin et al. 2018; Hung et al. 2010, 2012; Hwang et al. 2010; Jasani and Saks 2013; Kirklin et al. 2007; Klofutar et al. 2022; Klugman et al. 2011; Knight and Davies 2016; Lechner et al. 2024; Louw and Sanford-Dolly 2024; Park and Logsdon 2015; Poirier et al. 2020; Race et al. 2021; Slota et al. 2022; Star and Strickland 2008; Suzuki and Kurita 2025; Wolf et al. 2024; Wulfeck et al. 2024; Wyner and Doherty 2021; Yoneda et al. 2017; Yucel et al. 2025; Zakariya et al. 2025; Zhang et al. 2014)
Single measurement	18	(Beck et al. 2017; Boerwinkel et al. 2009; Chen et al. 2025; Cole et al. 2020; Donald and Barker 2016; Eberbach and Crowley 2017; Frøyland et al. 2016; He et al. 2019; Jarodzka et al. 2012; Kos et al. 2015; Monahan et al. 2019; Pellico et al. 2009; Qutieshat 2025; Reime et al. 2022; Shapiro et al. 2006; Tanaka et al. 2016; Terra and Black 2019; Yurumezoglu and Oztas Cin 2019)
Multiple sequential measurements	3	(Choi and Kim 2022; Colucci-Gray et al. 2025; Donald and Barker 2016)

Finally, three studies took multiple sequential measurements, assessing outcomes at multiple points during an intervention or following multiple sequential interventions (Table 2). Some of these studies conducted measurements in order to assess student growth during multistage interventions (e.g., a workshop series). For example, Colucci-Gray et al. (2025) assessed across a series of workshops. Workshop 1 was designed to introduce students to haptic input and using textures to make inferences about the natural world. Researchers assessed student discovery by quantifying how many textures they included in a clay sculpture based on this workshop. Workshop 2 asked students to use haptic input to identify trees using digital textures. In this workshop, students were documented to have done more “sense-making” with their observations, identifying patterns and differences between the trees. Here, researchers aimed to quantify how successful students were in their identifications with visual input alone versus with visual and haptic input. Finally, during Workshop 3, students were asked to conduct biodiversity counts of mosses, lichens, and invertebrates on their school grounds and researchers quantified the number of observations and identifications made by students. After this workshop, students asked more questions about how texture (form) related to organismal functions, suggesting a scaffolded advancement in their inquiry skills from more “everyday” to more “scientific” (Eberbach and Crowley 2009).

### Types of pedagogical method

#### Learning environment

Six papers included interventions associated with nontraditional learning spaces or modalities (e.g., virtual classrooms, outdoor spaces, live-action simulations), which we refer to as novel learning environments (Table 3; Kos et al. 2015; Yoneda et al. 2017; Race et al. 2021; Choi and Kim 2022; Klofutar et al. 2022; Reime et al. 2022). While representing a small subset of the total studies in this review, these studies included a range of age groups (prekindergarten to college) and a variety of countries of origin (United States, Norway, Japan, South Korea, and Slovenia). The average sample size was 60 participants (SD = 43.5). Half of these studies had fewer than 50 participants (Kos et al. 2015; Choi and Kim 2022; Reime et al. 2022) while the other three studies had 69, 88, and 129 participants (Yoneda et al. 2017; Race et al. 2021; Klofutar et al. 2022).

**Table 3:** Number of papers reviewed with interventions aligned with each of the following broad pedagogical methods.

**Type of pedagogical method**	**Number of included studies**	**References**
Learning environment	6	(Choi and Kim 2022; Klofutar et al. 2022; Kos et al. 2015; Race et al. 2021; Reime et al. 2022; Yoneda et al. 2017)
Strategies for scaffolding or supporting the observation process	19	(Alenezi 2020; Boerwinkel et al. 2009; Cole et al. 2015; Colucci-Gray et al. 2025; Eberbach and Crowley 2017; Frøyland et al. 2016; Hung et al. 2010, 2012; Hwang et al. 2010; Jarodzka et al. 2012; Knight and Davies 2016; Lechner et al. 2024; Louw and Sanford-Dolly 2024; Park and Logsdon 2015; Star and Strickland 2008; Suzuki and Kurita 2025; Tanaka et al. 2016; Wyner and Doherty 2021; Zhang et al. 2014)
Fine arts interpretation	24	(Agarwal et al. 2020; Beck et al. 2017; Chen et al. 2025; Cole et al. 2020; Fernandez et al. 2021, 2022; Ferrara et al. 2022; Ghorbani et al. 2025; Guo et al. 2021; Gurwin et al. 2018; He et al. 2019; Jasani and Saks 2013; Kirklin et al. 2007; Klugman et al. 2011; Lyon et al. 2013; Monahan et al. 2019; Pellico et al. 2009; Poirier et al. 2020; Qutieshat 2025; Shapiro et al. 2006; Slota et al. 2022; Wolf et al. 2024; Wulfeck et al. 2024; Yucel et al. 2025)
Integration of art creation (e.g., sketching, creative writing)	6	(Cole et al. 2015; Colucci-Gray et al. 2025; Donald and Barker 2016; Park and Logsdon 2015; Yurumezoglu and Oztas Cin 2019; Zakariya et al. 2025)

Two studies tested the influence of field experiences on students’ observation skills (Kos et al. 2015; Choi and Kim 2022). Choi and Kim (2022) implemented repeated field excursions for 6th grade students aimed at improving rock identification skills (treatment group only) and analyzed student drawing and writing to determine if the observation skills demonstrated were “everyday,” “transitional,” or “scientific” (according to the framework by Remmen and Frøyland 2020, which is a modification of the framework by Eberbach and Crowley 2009). Choi and Kim (2022) collected repeated measurements over nine field trips and found that students in the control group displayed “everyday” and “transitional” observations while the field-trip group showed more advanced “transitional” and “scientific” observations. Kos et al (2015) also investigated the value of field experiences by exposing the treatment group to field trips in their local forest. However, their results were mixed, potentially due to experimental design issues. They found that children who received outdoor education improved significantly for some tasks relative to the control group (naming leaves and connecting fruits or cones to the corresponding leaf), but results were inconclusive for other metrics (matching leaves on cards and their physical specimens). Because students were exposed to the same activities during the pre- and posttest, the authors suspect that the lack of significant differences for some tasks was due to familiarity with the tasks.

Additionally, two studies (Race et al. 2021; Klofutar et al. 2022) assessed how content delivery modality affects observation skill building. Klofutar et al. (2022) conducted instruction indoors using “vicarious” teaching tools such as games, books, and videos for a group of preschool students and compared their observation skill outcomes to a second “outdoor instruction” group. They found that while both groups’ observation skills improved, the outdoor learning group showed higher increases and longer retention of skills compared to the “vicarious” group. This effect was partially attributed to students’ ability to incorporate feedback from multiple senses during observation in the outdoor setting. Importantly, Klofutar et al. (2022) also state that their study demonstrates the value of short field experiences for improving students’ scientific reasoning, with their study including 5 h long lessons for each group of students. Race et al. (2021) investigated how online learning for an introductory undergraduate field course during the COVID-19 pandemic compared to in-person learning during previous years. Interestingly, despite some reductions in community building in the online class, Race et al. (2021) noted qualitative trends and survey responses indicating that online students’ self-efficacy in observation skills showed greater improvement. For example, their survey results showed that weekly observation tasks “increased [students’] ability to recognize patterns in nature, and be better observers of their space” with one student commenting that “now I feel like when I visit a space I can visualize deeper interactions that are going on” (Race et al. 2021). Generally, the authors concluded that online field courses can provide comparable benefits to in-person field courses.

Finally, two studies assessed the use of simulations and their effects on observation competency (Yoneda et al. 2017; Reime et al. 2022). One study found that simulations of observable hazards (in this case, hazards in healthcare settings) improved observation frequency in students receiving observation training (Yoneda et al. 2017). A second study with simulated hazards found that training in groups, rather than as individuals, increased the total number of observed hazards (Reime et al. 2022).

#### Observation process scaffolding and support

Of the 53 studies reviewed, 19 contained interventions that provided scaffolding or supportive tools for making observations (Alenezi 2020; Boerwinkel et al. 2009; Cole et al. 2015; Eberbach and Crowley 2017; Frøyland et al. 2016; Hung et al. 2010, 2012; Hwang et al. 2010; Jarodzka et al. 2012; Knight and Davies 2016; Lechner et al. 2024; Louw and Sanford-Dolly 2024; Park and Logsdon 2015; Star and Strickland 2008; Suzuki and Kurita 2025; Tanaka et al. 2016; Wyner and Doherty 2021; Yurumezoglu and Oztas Cin 2019; Zhang et al. 2014). These papers included a range of age groups (prekindergarten to college and medical school) and a wide range of countries of origin (Taiwan, Turkey, Japan, South Korea, Denmark, Norway, United States, Scotland, Germany, Netherlands, and Saudi Arabia). Interestingly, these studies represent most of the disciplines in Table 1, including hydrology, biology/ecology, health science, astronomy, design, teacher training, and geology. The mean sample size for studies within this category was 102 participants (SD = 137.8). However, nearly half of these studies had fewer than 50 participants (Frøyland et al. 2016; Hung et al. 2010, 2012; Hwang et al. 2010; Louw and Sanford-Dolly 2024; Park and Logsdon 2015; Star and Strickland 2008; Suzuki and Kurita 2025; Tanaka et al. 2016), four studies had over 100 participants (Cole et al. 2015; Knight and Davies 2016; Wyner and Doherty 2021; Zhang et al. 2014), and one study notably did not state a sample size (Yurumezoglu and Oztas Cin 2019).

The studies in this category fell into three main groups based on the goals of scaffolding: (1) to help students better apply the steps of the observation process (Alenezi 2020; Eberbach and Crowley 2017; Frøyland et al. 2016; Lechner et al. 2024; Star and Strickland 2008; Wyner and Doherty 2021); (2) to highlight important identifying or diagnostic features for students (Hwang et al. 2010; Jarodzka et al. 2012; Knight and Davies 2016; Louw and Sanford-Dolly 2024; Park and Logsdon 2015; Suzuki and Kurita 2025; Tanaka et al. 2016; Zhang et al. 2014); or (3) to help students make inferences about patterns or processes based on their observations (Boerwinkel et al. 2009; Cole et al. 2015; Hung et al. 2010, 2012; Yurumezoglu and Oztas Cin 2019).

Studies that focused on scaffolding the steps of the observation process tested the effects of explicitly explaining the steps of scientific observation during workshops or lessons. These skills were taught alongside discipline-specific knowledge (e.g., rock identification, patient diagnosis, river classification) and the scaffolding was applied in several ways. For example, Lechner et al. (2024) examined the effectiveness of worked examples for improving students’ application of the steps of the observation process when classifying river hydromorphology. They found that students who completed worked examples in class were better able to apply the individual steps of observation. Similarly, Alenezi (2020) worked through a series of observation exercises with students prior to conducting patient role-playing scenarios and found “highly significant” improvements in disciplinary knowledge and observation skills post-intervention. Both Wyner and Doherty (2021) and Frøyland et al. (2016) applied “curriculum grounded in scientific observation practice” that aimed to support a more process-based understanding of tree and rock classification based on an understanding of the steps of scientific observation. In both cases, students demonstrated better classification skills and improved retention of identification skills. Frøyland et al. (2016) additionally found that students who received scientific observation-based teaching were more likely to engage in process-based thinking rather than simple memorization for rock classification. Finally, Star and Strickland (2008) and Eberbach and Crowley (2017) found that less formal interventions such as asking guiding questions (e.g., “What did you notice?”) also improved participants’ observation skills. Notably, this was true for both young children (Eberbach and Crowley 2017) and teachers in training (Star and Strickland 2008) and is in line with other studies in this review which suggest that the process of communicating observations verbally, in writing, or through art can improve observation skills.

Studies which tested scaffolding strategies that highlighted relevant features for participants typically focused on either written instructions (e.g., dichotomous keys) or visual cuing (e.g., expert eye-tracking overlaid on video). Knight and Davies (2016) assessed the use of a mobile dichotomous key in a museum setting and found it improved the “level of detail students provided and the number of scientific terms they used” compared with students who received an instructor-led tour of the museum. Park and Logsdon (2015) instead asked students to write their own instructions for how to construct a 3D model that each student made using blocks. They found that reviewing other students’ instructions and providing feedback resulted in students better identifying important details for inclusion in future instructions, and as a result their observation and descriptive writing skills improved with multiple iterations of this exercise.

Three studies showed students videos which highlighted relevant features (Jarodzka et al. 2012; Suzuki and Kurita 2025; Tanaka et al. 2016), including two which applied expert gaze tracking to the video or circled relevant features to advance diagnostic skills (Jarodzka et al. 2012; Suzuki and Kurita 2025). The results from Jarodzka et al. (2012) were the most conclusive, emphasizing the utility of not only circling relevant features, but also blurring out irrelevant features in such video training tools. A related study by Hwang et al. (2010) utilized a "critical-feature-finding algorithm” to support students in observing commonly misrecognized features of plants. This decision-tree guidance mechanism positively influenced students’ motivation to observe in a butterfly garden. Louw and Sanford-Dolly (2024) combined these approaches by testing the efficacy of training with macroinvertebrates.org, an online aquatic macroinvertebrate identification tool that pairs written identifying features for species with zoomed images of those features.

One study in this subcategory focused more on removing obstacles to observation through the use of an artificial reality software that reduced the impacts of cloud cover and equipment challenges on students’ ability to observe astronomical phenomena (Zhang et al. 2014). Zhang et al. (2014) found that the use of artificial reality technology significantly improved the performance of astronomical observation skills such as constellation identification.

Studies which provided scaffolding to help students make connections or draw inferences based on their observations applied a variety of techniques. For example, Hung et al. (2012) used digital concept maps to help students make inferences about connections between ecosystem components. Additionally, Hung et al. (2010) created and tested scaffolded digital worksheets intended to help students move from guided observation through autonomous observation and finally to independent inquiry during a series of field trips. Both studies documented increases in student observation skills post-intervention quantified through performance on the Computerized Ecology Observation Competence Assessment (Hung et al. 2012) or the number of autonomous and relevant observations students made about the wetlands they were visiting (Hung et al. 2010). Cole et al. (2015) emphasized the importance of observation record-keeping as a method of supporting observation synthesis through the application of structured “moon journals.” They found that students who had more entries total, more types of entries (e.g., drawing, writing, measurements), and more quality entries performed better on knowledge tests and spatial reasoning tests after the moon journaling intervention. Lastly, Boerwinkel et al. (2009) used the perspective of form and function in biology and design (“the designer’s glasses”) to support student observations by providing them with a framework through which to view specific features. The study found that students were more motivated to investigate the form of specific features when they began by exploring the function of those features.

#### Arts-based interventions to strengthen observation skills

Of the studies reviewed, 30 investigated arts-based activities and training as tools for developing observation skills (Table 3). Twenty four studies assessed art interpretation, usually using visual thinking strategies, to practice observation skills first in the context of noticing details in pieces of art, and then translating those observation skills to clinical settings. The remaining six papers described activities and lessons that involved creating art to foster more detailed observation, for example, with students making repeated illustrations of the human body (Lyon et al. 2013).

##### Fine arts interpretation

**Fig. 2:**
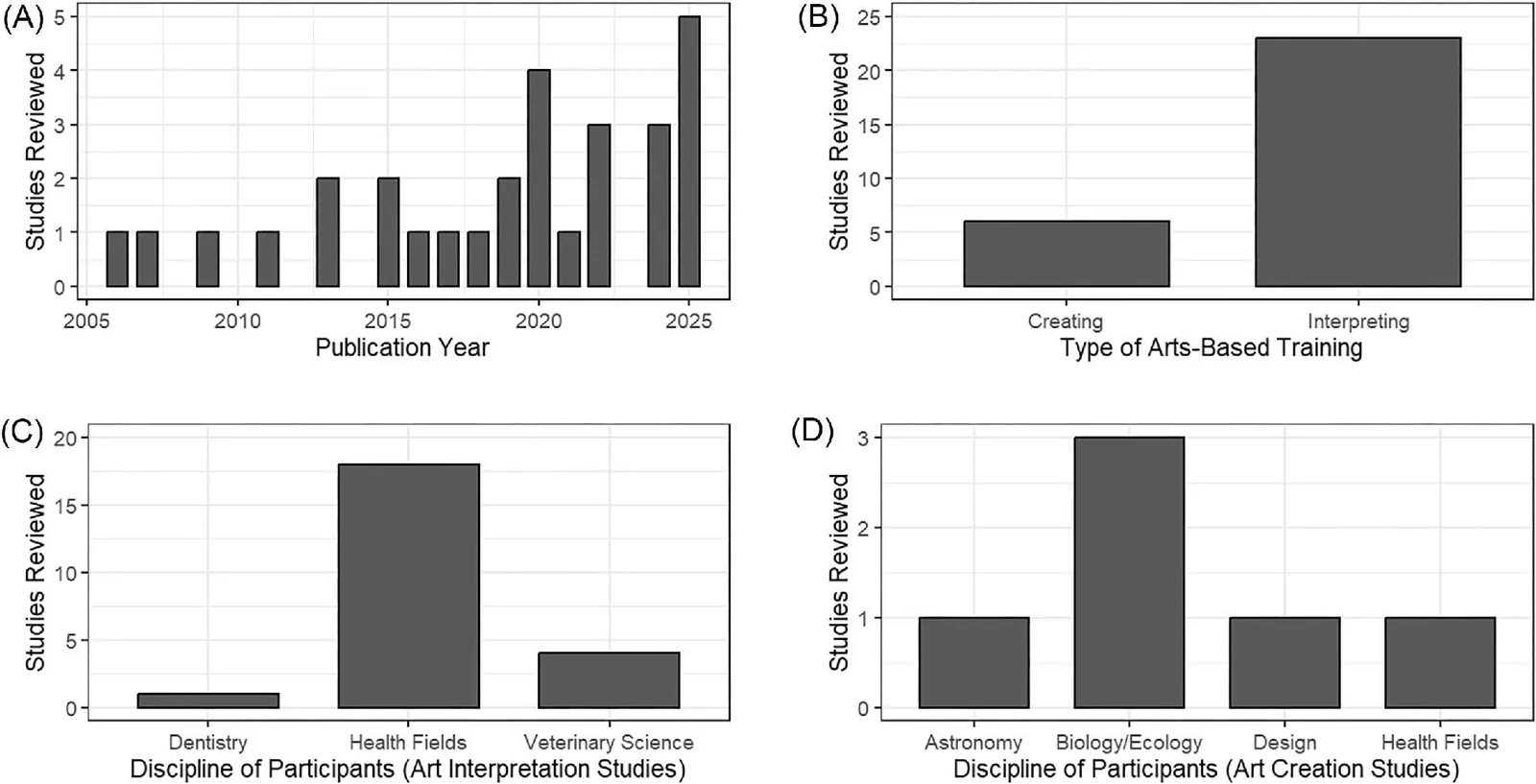
Characteristics of studies that fell in the “arts-based interventions” category of pedagogical tools, including: (**A**) frequency of publication years for arts-based studies, (**B**) frequency of art creation and art interpretation interventions, (**C**) frequency of studies from different disciplines that employed art interpretation interventions, and (**D**) frequency of studies from different disciplines that employed art creation interventions.

Fine arts interpretation was the most common pedagogical method reviewed. Most of the papers included in this category were published between 2019 and 2025 (Fig. 2). Sample sizes ranged from 4 to 333, with a mean sample size of 68 students (SD = 63.8). The age of participants in all 24 papers in this category fall into the college + age group, and the majority of studies were conducted with medical students (Fig. 2). None of the studies reviewed came from biology or ecology disciplines. We included arts interpretation studies from 12 different countries.

Interestingly, the vast majority of the fine arts interpretation papers reviewed (21 of 24) resulted in either increased observation skills as measured by increased numbers of observations, greater numbers of possible diagnoses proposed, increased alignment with expert diagnostic assessments (Agarwal et al. 2020; Beck et al. 2017; Chen et al. 2025; Fernandez et al. 2022; Ferrara et al. 2022; Guo et al. 2021; Jasani and Saks 2013; Kirklin et al. 2007; Klugman et al. 2011; Pellico et al. 2009; Poirier et al. 2020; Slota et al. 2022; Wolf et al. 2024; Yucel et al. 2025), or increased perception of self-efficacy in observation skills (Jasani and Saks 2013; Beck et al. 2017; He et al. 2019; Monahan et al. 2019; Cole et al. 2020; Poirier et al. 2020; Ferrara et al. 2022; Qutieshat 2025). Several of these further documented retention of observation skills (Beck et al. 2017; Fernandez et al. 2022; Guo et al. 2021; Wulfeck et al. 2024). While most of these studies took place in a lecture/workshop format (Beck et al. 2017; Fernandez et al. 2022; Ferrara et al. 2022; Ghorbani et al. 2025; Jasani and Saks 2013; Kirklin et al. 2007; Monahan et al. 2019; Poirier et al. 2020; Qutieshat 2025; Shapiro et al. 2006; Slota et al. 2022; Wolf et al. 2024; Wulfeck et al. 2024; Yucel et al. 2025) rather than in a museum setting (Agarwal et al. 2020; Ferrara et al. 2022; Guo et al. 2021; Gurwin et al. 2018; He et al. 2019; Klugman et al. 2011; Pellico et al. 2009), improvements in observation skills were seen regardless of setting. Similarly, improvements were observed for a variety of intervention durations (e.g., 10–15 min per week vs. a 12-week art evaluation course). However, the shorter duration interventions sometimes had less conclusive results (Fernandez et al. 2021; Poirier et al. 2020; Wolf et al. 2024).

Additionally, while one study emphasized the utility of art interpretation training in supporting clinical applications by developing “critical looking” and causing students to think more about relevant features (Lyon et al. 2013), two studies highlighted the importance of clinical knowledge and context in art interpretation training. Shapiro et al. (2006) found that clinical and art-based training resulted in development of different dimensions of observation. For example, their results showed that for students who received only clinical training, “the ‘meaning’ of patterns pointed to clinical schema and differential diagnosis.” Whereas in the art and dance training treatments, “‘meaning’ had more to do with understanding the human condition.” They further found that the observation skills gained in the clinical training better supported multiple dimensions of pattern recognition in a disease context. While these results suggest that art-based interventions might support more holistic observation skill development, clinical knowledge and context is critical for applying observation skills in medicine. Similarly, Fernandez et al. (2021) applied art interpretation training and found that students significantly improved at observing fine art following the intervention, but that these improvements did not extend to cytological images. Importantly, while many of the studies assessed students’ observation ability with radiographs or clinical images (Agarwal et al. 2020; Beck et al. 2017; Chen et al. 2025; Cole et al. 2020; Fernandez et al. 2021, 2022; Ferrara et al. 2022; Ghorbani et al. 2025; Guo et al. 2021; Gurwin et al. 2018; Jasani and Saks 2013; Kirklin et al. 2007; Qutieshat 2025; Wolf et al. 2024; Wulfeck et al. 2024; Yucel et al. 2025), only one study assessed how observation skills gained using art interpretation translated to assessment of “standardized patients” (Monahan et al. 2019). Further, this study only assessed students’ perceptions of self-efficacy in observation skills; more research on the relationship between art interpretation training and in-person diagnostics could be a critical area of future research.

Finally, three studies assessed the influence of art interpretation training on empathy (He et al. 2019; Slota et al. 2022) or ambiguity tolerance (He et al. 2019; Klugman et al. 2011). Both of these characteristics were found to increase following art interpretation training, which He et al. (2019) links to the development of a more “humanistic view of medicine that advocates an unbiased and compassionate approach to others” which focuses more on patient narrative reconstruction, and the ability to be more open to multiple explanations for diagnostic cues.

##### Art creation

Only six studies included in this review integrated art creation and they were all published between 2015 and 2025. The number of participants in these studies ranged from 9 to 333, with a mean of 103 (SD = 132) and one unknown sample size (Yurumezoglu and Oztas Cin 2019). The age of participants in these studies ranged from preschool to college+, with half of the studies taking place at the primary school level (Fig. 2). The studies came from five different countries of origin, including two from the United States and one each from Malaysia, South Africa, Scotland, and Turkey. Notably, none of the studies in this category had a control group. Four studies related to making observations in biology/ecology (Colucci-Gray et al. 2025; Donald and Barker 2016; Yurumezoglu and Oztas Cin 2019; Zakariya et al. 2025), one related to astronomy (Cole et al. 2015), and one had mixed applications (Park and Logsdon 2015).

Both the artistic medium and the outcomes assessed varied widely among these studies. Three studies included student sketching or drawing (Cole et al. 2015; Yurumezoglu and Oztas Cin 2019; Zakariya et al. 2025). Zakariya et al. (2025) explored the impacts of guided nature journaling with annotated sketching of the natural world, finding that participants felt greater connection to nature and higher confidence in observation skills after completing the activities. The interventions presented by Cole et al. (2015) and Yurumezoglu and Oztas Cin (2019) included many other aspects of observation and note-taking, so the specific impact of the sketching was unclear. One small study focused on the impact of writing poetry about animals, with the majority of students reflecting that this activity was useful and complementary to previous work on writing concise scientific descriptions (Donald and Barker 2016). In another study, secondary students built 3D objects using commercially available building toys, while trying to observe their work and write accurate directions for someone else to create the same object (Park and Logsdon 2015). Engaging in this combination of building, self-reflective observation, and writing yielded increases in both self-perceived observation skills and rubric scores for the quality of student building instructions (Park and Logsdon 2015). Finally, one study described student work with clay; students created fantasy clay animals and added textures using materials collected from the school grounds (Colucci-Gray et al. 2025). When reflecting on their animals, students were able to draw inferences from between animal textures or shapes and an animal’s behavior and ecology (Colucci-Gray et al. 2025).

## Discussion

We identified a diverse set of 53 studies with evidence for pedagogical practices which might be useful for building observation skills in undergraduate integrative or organismal biology courses. These studies spanned 10 academic disciplines, with most assessing undergraduate or postgraduate students. Interestingly, the majority of these studies were published in the last 10 years, suggesting that this is an emerging field of study. Only 13 studies included both a control group and assessed outcomes both before and after the intervention. While over 25% of the studies reviewed were in biology fields, the majority of the reviewed studies were from disciplines outside of integrative and organismal biology. However, many of the interventions studied in other disciplines could be highly applicable in integrative and organismal biology courses and offer several insights into undergraduate integrative and organismal biology teaching. Based on this review, below we summarize practices for developing students’ observation skills in integrative and organismal biology, synthesizing practices from biology classrooms as well as practices researched in other fields that have high potential for translation to integrative and organismal biology courses.

In this review, we found a small body of research on ways in which novel learning environments can support the development of observation skills in integrative and organismal biology. Observation skill studies conducted in integrative and organismal biology fields showed benefits for student learning both in more traditional settings such as group field trips (Klofutar et al. 2022) as well as nontraditional experiences such as online learning (Race et al. 2021) and “vicarious learning” through games, videos, and books (Klofutar et al. 2022). Another form of vicarious learning, real-world simulation, can be effective in developing observation skills in medical fields (Reime et al. 2022; Yoneda et al. 2017). These findings suggest that virtual or artificial versions of environments with observable phenomena could be useful tools for students with limited access to field experiences or specimens. While we found no studies specifically applying this methodology to integrative and organismal biology fields, simulations might be a relevant tool for understanding a variety of ecological, physiological, and behavioral phenomena. Indeed, two recent reviews highlight the value in simulation-based learning in biology education (Byukusenge et al. 2022; Robledo and Prudente 2021), but note that most of the simulations available focus on abstract or microscale concepts (e.g., evolution, genetics, cell biology, with some from anatomy). Additionally, these reviews did not include any studies that explicitly tested the value of simulations in biology education for improving observation skills. As a result, future research on the effectiveness of simulation-based learning for observation skill development in ecology, physiology, and animal behavior could be fruitful. Finally, several studies on outdoor biology education noted the importance of integrating several senses into observation experiences (Colucci-Gray et al. 2025; Klofutar et al. 2022), suggesting that multisense “noticing” can result in more accurate observations and greater persistence of skill improvements. Importantly, these studies highlight the diversity of senses that can be used in observation instruction, offering avenues for instructors to reduce barriers and diversify pedagogical strategies to make learning accessible for all students.

A variety of scaffolding techniques also supported the development of observation skills in other disciplines and could easily be translated to integrative and organismal biology. For example, scaffolding the observation process by explicitly walking students through the steps of the observation process in the context of disciplinary work (e.g., through worked examples as in Lechner et al. 2024, or ask guiding and probing questions throughout the observation process as in Eberbach and Crowley 2017; Star and Strickland 2008) can result in more detailed and accurate observations. Some scaffolding tools which are commonly used in integrative and organismal biology to highlight relevant features (e.g., dichotomous keys) can be useful for helping students learn to “notice” relevant features and disregard irrelevant features (Knight and Davies 2016). Work from medical disciplines where researchers blurred irrelevant features further significantly improved students’ ability to notice relevant features (Jarodzka et al. 2012). Louw and Sanford-Dolly (2024) applied similar techniques for aquatic macroinvertebrate identification with macroinvertebrates.org, an online identification tool that pairs written identifying features for species with zoomed images of those features. The production and application of similar online identification tools for other taxa might be useful both for learning species identification as well as providing students with a framework for noticing relevant features and applying them to concepts in integrative and organismal biology (i.e., thinking about adaptations of organisms based on which features differentiate them from other species). In fact, scaffolding which aims to bridge the “noticing” and “expectations” aspects of the observation process would offer great benefits for integrative and organismal biology pedagogy. The studies reviewed here demonstrated that scaffolded worksheets and concept maps that support synthesis of observations and connection to biological concepts and processes supported more autonomous observation and inquiry (Hung et al. 2010, 2012). These findings emphasize that more “scientific” observation arises from intentional engagement with the steps of the observation process, support in understanding what features are relevant in a given discipline, and activities that bridge the gap between noticing relevant features and applying them to relevant concepts.

### Potential for arts-based interventions in undergraduate biology education

We found a large body of literature supporting the use of arts-based interventions to improve observation skills, with strong potential for translation to integrative and organismal biology courses. Training and practice in fine arts interpretation, the most common pedagogical method we reviewed, has been shown to increase the number and accuracy of observations (e.g., Agarwal et al. 2020; Beck et al. 2017) and increase perception of self-efficacy and retention of observation skills (e.g., Cole et al. 2020; Ferrara et al. 2022). Although all of the arts interpretation studies were applied to medical contexts, more detailed and accurate observations and greater retention of observation skills can all translate to more effective scientific observations and scientific observers in the broader field of organismal and integrative biology. For example, exercises that teach students how to make more detailed and accurate observations could help them identify species, draw comparisons between species, relate observations to ecological and evolutionary processes, identify patterns across a set of observations, synthesize observations, and create more detailed and relevant observation records.

However, the ideal approach to implementing arts interpretation in a biology context is not entirely clear. With visual thinking strategies, experts facilitate discussions of art, first prompting the students to notice (e.g., with the question “What’s going on in this picture”), then encouraging a connection between noticing and expectations (“What do you see that makes you say that?”), before guiding students to iterate through this process (“What more can we find?”; Yenawine 2013). In an organismal biology course, students may benefit from the direct application of this approach, assuming the instructor has developed skills to lead a critical discussion of a piece of visual art. On the other hand, this approach to noticing and questioning could be adapted to photographs, videos, or live observations of living organisms. Whether these approaches benefit noticing skills like describing or classifying organisms, expectations skills like making ecological, behavioral, or evolutionary predictions about an organism, or improve productive dispositions, students may benefit in course performance, engagement, and career skills. However, it is important to note that some studies (Shapiro et al. 2006, Fernandez et al. 2021) emphasized the importance of discipline-specific knowledge and context for supporting discipline-relevant observation skills when employing fine art interpretation training. These studies suggest that providing scaffolding which helps translate observation skills built through fine arts interpretation into a discipline-specific context might be important.

Interestingly, two studies from health sciences highlighted increases in ambiguity tolerance in students who received fine art interpretation training (He et al. 2019; Klugman et al. 2011). Two recent reviews discuss the importance of ambiguity tolerance in education, stating that it is a core competency in science education and development as well as language learning (Kianinezhad 2024; Vogel and Hunecke 2024). Ambiguity tolerance is a students’ capacity to handle uncertainty and can be a prerequisite for being able to explore creative explanations and outcomes and integrate knowledge. As a result, development of this skill could be important for knowledge acquisition and inquiry-driven learning in integrative and organismal biology which encompasses a plethora of fields that are defined by complex patterns, concepts, and interactions that can be difficult to interpret. In the context of scientific observations, ambiguity tolerance could be an important element of productive dispositions which in turn allows students to move from noticing to expectations by enhancing their ability to hold multiple possible explanations as they synthesize and make sense of observations. Studies which explicitly assess how a variety of interventions influence students’ ambiguity tolerance in integrative and organismal biology applications is a clear area of future research.

Despite the small number of art creation studies included in this review, these studies connect to some common practices in organismal biology education. The studies included student sketching or drawing (Cole et al. 2015; Yurumezoglu and Oztas Cin 2019; Zakariya et al. 2025), writing poetry (Donald and Barker 2016), building three-dimensional objects (Park and Logsdon 2015), and working with clay (Colucci-Gray et al. 2025). The overarching theme for the studies in this category is that the practice of creating art encourages students to slow down, make closer observations, and notice more deeply. Notably, it was the practice itself of creating, not the product, that led to better observations. It is perhaps surprising that integration of art creation is among the least common pedagogical methods we came across in our review, considering the widespread use of field and lab notebooks in the integrative and organismal biology fields. It is less surprising, however, that this method had the highest proportion of studies in the biology/ecology field. Sketching or drawing, for example, is a method that could help students draw comparisons between species, create more detailed and relevant observation records, and synthesize and identify patterns across a set of observations; all of which are important skills for biologists. Additionally, sketching and drawing exercises could easily be applied and adapted to a variety of topics, learning environments, and age groups. More work is needed to identify optimal approaches to guiding this sketching for biology undergraduates.

While most of the studies in this review were not conducted in integrative and organismal biology fields, many of the findings could be applicable to biology courses. To support this translation, we have developed a table addressing how each of the four categories of interventions might be applied to biology classrooms and what the expected outcomes might be with regard to the development of observation skills (Table 4).

**Table 4:** Examples of how to apply each of the four pedagogical approaches shown to improve observation skills to integrative and organismal biology courses.

**Pedagogical approach**	**Examples from the literature**	**Examples of applying this approach to an IOB course**	**Anticipated outcome based on literature**
Manipulating the learning environment	Outdoor field trips, online classroom, simulations	Taking students outside a few times throughout the quarter to make observations using all their senses. (Does not have to be a long excursion.) Using videos of wildlife and habitats to simulate field observations.	Increased frequency and accuracy of observations and increased retention of observation skills.
Scaffolding the observation process	Workshops or lessons that explicitly explain the steps of observation, using tools or cues that highlight important diagnostic or identifying features of organisms, using tools or cues to guide student’s inferences about patterns or processes	Instructor or expert can walk through the steps they follow when making an observation. For example, if identifying a bird, do you start by noticing its size, color, shape, sound? Or, practice making observations together as a class with instructor guidance. For example, show a picture or video of an organism or habitat and talk through the observation process. Highlight important identifying features for students by providing a dichotomous key. Help students infer patterns and make connections to processes by asking guiding questions: What do you notice? What did you observe that led you to this conclusion?	More detailed and accurate observations that focus on key diagnostic or identifying features. Students may also feel stronger motivation and confidence to make further observations and inferences.
Engaging in arts interpretation	Practicing observation skills first in the context of noticing details in pieces of art, and then translating those observational skills to clinical contexts	Inviting an expert in art interpretation to lead a class activity where students practice observing details in a piece of art. Then ask students to apply these skills to the observation of an animal specimen or field observation. A biology instructor could try translating this strategy by first showing an image of an organism and helping students notice details in the image. They could then show a preserved specimen, video, or new image of an organism and ask the students to make observations independently.	More numerous, detailed, and accurate observations after the training.
Engaging in arts creation	Creating art (e.g., poetry, sketches) during the process of observation	Sketch museum specimens to compare key features. Go outside and paint the same flower every week as it grows and senesces. Show students a video of an interesting animal behavior and ask them to write a poem about what they observe.	Greater connection to nature, increased confidence in observation skills, and more accurate inferences.

### Future research directions

It is important to note that while there was a dearth of papers specifically assessing how the pedagogical techniques reviewed here affect students’ observation skills in integrative and organismal biology courses, that does not mean that these pedagogical techniques are not widely used in these courses. For example, it is well known that nature journaling and specimen sketching were foundational practices for natural historians and early organismal biologists (Belknap 2018; Merkle et al. 2020). While these practices are still frequently integrated into integrative and organismal biology courses, Merkle et al. (2020) suggests that competency in this skillset is declining. Further, much of the pedagogical research on art-based techniques in integrative and organismal biology focuses on student perceptions about and connection to nature (e.g., Treibergs et al. 2022). This leaves a substantial gap in the literature regarding best practices for the implementation of art-based learning for the development of observation skills in biology classrooms, a gap that is mirrored in all four of the pedagogical categories in this review. Clearly, more work is needed to identify optimal approaches to guiding these art-based approaches to support the development of observation skills for biology students. We suggest that partnerships between biology and education faculty to more systematically study the tools which are already being used in biology classrooms in a variety of age groups could be extremely fruitful for advancing this area of knowledge and improving the development of observation skills for students. For example, while their study does not directly quantify effects on observation skills and only assesses one student, Hayes and Stephens (2026) provide a framework for how a researcher might assess the efficacy of nature journaling in improving the quantity and quality of student engagement with “cross-cutting concepts” through time, several of which relate strongly to observation skills in integrative and organismal biology (e.g., pattern identification and understanding the relationship between structure and function). Alternatively, according to many of the studies in this review, even collecting data on more basic metrics such as the number of observations made or students’ self-assessment of observation efficacy following a pedagogical intervention could be extremely valuable.

Additionally, research that assesses observation-focused pedagogy specifically for undergraduate biology students is lacking. Among the biology and ecology studies included in this review, only two studies focused on participants of college age or older. Fundamental aspects of noticing and expectations likely translate across all age groups, but the academic tasks that college biology students regularly complete may consistently differ from those of primary and secondary school students. For example, college biology courses particularly emphasize expectation skills like integrating ecological and evolutionary theory with observations to interpret patterns and make new predictions (Russell 2009). The degree to which these skills first require strong noticing skills, and how to best build both scientific noticing and expectations, may be a productive avenue for pedagogical research on biology undergraduate observation skills.

### Limitations

Although this literature review identified studies across a diversity of methods and disciplines, we struggled to define observation skills in a way that was both precise and inclusive of observation across many disciplines. We suspect that observation skills may be referred to with several other terms that we did not detect in our scoping, so it is likely that we have missed relevant studies within each of our intervention categories, and there may be entire other types of intervention that did not appear in our search results. In addition, the exact teaching context of the interventions in studies was not always clear, both because of our inexperience with typical course structures in countries outside of the United States and because information on institution types and class size or structure (e.g., small primarily undergraduate institution versus large research institution, courses for majors versus nonmajors, courses with mostly passive lecture versus with substantial active learning) was sometimes absent or incomplete in published study methods. The feasibility and potential impact of particular pedagogical interventions may vary greatly depending on course and institution context.

## Conclusions

This review summarized a diverse suite of studies that can inform the teaching of observation skills for undergraduates studying integrative and organismal biology. Pedagogical strategies include manipulating the learning environment, scaffolding the observation process, and engaging with arts interpretation or creation. Arts interpretation shows particular value for learning observation skills in health fields, and may translate effectively to skills for observing biological phenomena. However, more work is needed to implement and systematically assess observation skills pedagogy in an undergraduate biology context, ideally with intervention and control groups and pre-post measurements of observation skills.

## Supplementary Material

obag037_Supplemental_File
