# Supplementary material for: Strategies to Build Observation Skills for Integrative and Organismal Biology Undergraduates: A Scoping Review
Source: Integr Org Biol. 2026 Jul 13;8(1):obag037. doi: 10.1093/iob/obag037 (PMC13416737; doi:10.1093/iob/obag037)
Supplement: obag037_Supplemental_File [file obag037_supplemental_file.docx]

## Supplemental Materials

Supplemental table S1. Search queries and number of results.

| **Query** | **# Results** |
| --- | --- |
| ("scientific observation" OR "science observation" OR "science" OR STEM OR natur* OR biolog* OR ecolog*) AND (teach* OR instruct* OR train* OR pedagog* OR learn* OR build* OR develop* OR foster*) AND ("observational competence" OR "observation competence" OR "observational competency" OR "observation competency" OR "observation skills" OR "observational skills" OR "nature journal") | 146 |
| ("scientific observation" OR "science observation" OR "nature journaling" OR "observation skills" OR "observational skills" OR "observational competence" OR "observation competence" OR "observational competency" OR "observation competency" OR "field notebook*" OR "nature journal*") AND (teach* OR instruct* OR pedagog* OR train* OR learn* OR develop* OR foster*) AND (assess* OR measure* OR evaluat* OR rubric* OR test* OR instrument*) AND ( science OR STEM OR nature OR natural OR student*) | 504 |
| ("scientific observation" OR "science observation" OR "systematic observation" OR "nature journaling" OR "science" OR "environmental science" OR STEM OR natur*) AND (teach* OR instruct* OR train* OR pedagog* OR learn* OR build* OR develop* OR foster*)) AND ("observational competence" OR "observation competence" OR "observational competency" OR "observation competency" OR "observation skills" OR "observational skills") AND ("medicine" OR "clinical" OR "veterinary" OR "ecology" OR "biology" OR "hydrology" OR "identification" OR "geology" OR "nature" OR "museum" OR "specimens") | 77 |
| ("scientific observation" OR "science observation" ) AND (teach* OR instruct* OR train* OR pedagog* OR learn* OR foster* OR "worked examples") | 227 |
